# Temperature-Dependent Kinetic Parameters for the Alkaline Oxygen Evolution Reaction on NiFeOOH

**DOI:** 10.1021/acsenergylett.5c01387

**Published:** 2025-06-03

**Authors:** Onno van der Heijden, Rafaël E. Vos, Marc T. M. Koper

**Affiliations:** 4496 Leiden Institute of Chemistry, Leiden University, 2333 CC Leiden, The Netherlands

## Abstract

Kinetic parameters of the oxygen evolution reaction on NiFeOOH remain elusive. Here, we studied the temperature dependence of this reaction to extract kinetic parameters, such as the temperature-dependent Tafel slopes, transfer coefficients, (standard) apparent activation energies, and pre-exponential factors. We observe a linear increase in the Tafel slope (∼30 mV/dec at room temperature) with increasing temperature when nonkinetic effects were excluded, whereas the trend reverses when significant nonkinetic effects were involved. Standard apparent activation energies of ∼75 kJ/mol were found. This has to be interpreted with regards to the mechanism containing two electrochemical presteps prior to a chemical rate-determining step (EEC or E^2^C) and consists mostly of the free energy of the pre-equilibrium steps. The apparent activation energy at an overpotential of 0.265 V then decreased to ∼13 kJ/mol. Therefore, temperature-dependent studies can provide important mechanistic insights into electrocatalytic reactions, provided care is taken that nonkinetic effects are eliminated.

## Graphical abstract

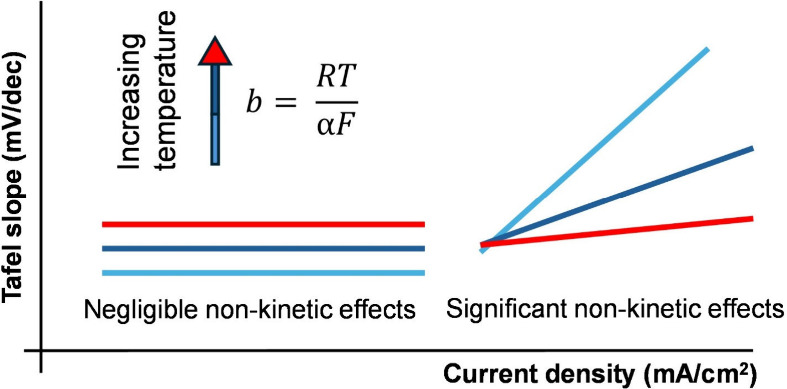

To understand the kinetics of electrocatalytic reactions, it is critical to extract kinetic parameters, such as the Tafel slope and corresponding transfer coefficients. However, it is difficult to determine the specific mechanism from a single Tafel slope value at room temperature. This is particularly true for multielectron transfer reactions, for which the same Tafel slope may allow different interpretations, depending on the surface coverage.^1–3^ This is further complicated by nonkinetic effects (such as mass transport and bubble formation), although such effects can be minimized using thin layers and fast mass transport conditions.^4–10^ To evaluate the influence of nonkinetic effects, a Tafel slope plot can be constructed, in which the Tafel slope is calculated over small potential regions and plotted vs the average *E* or *J* (or by differential Tafel analysis). In such a plot, a horizontal, or constant, Tafel slope region would indicate a kinetically meaningful value.^4,5,11^ Moreover, the Tafel slope characteristics can be studied as a function of the temperature. According to the Butler–Volmer equation at large overpotential, a linear increase in the Tafel slope with temperature is expected if the (effective) transfer coefficient is temperature independent:

1 $$b=\frac{RT}{\alpha F}$$

where *b* is the Tafel slope, *R* is the universal gas constant, *T* is the absolute temperature, α is the effective transfer coefficient, and *F* is Faraday’s constant.^3^

Conway has reviewed the temperature dependence of electrochemical reaction rates.^12^ He and his co-workers studied the Tafel slope behavior and transfer coefficients for the Hydrogen Evolution Reaction (HER) on mercury drop electrodes and suggested that the total transfer coefficient consists of an enthalpic component (*α*_*H*_) and an entropic component (*Tα*_*S*_).^13^ In studies of the HER, large variations in the enthalpic and entropic transfer coefficients and therefore the temperature dependence of the Tafel slope were found for different materials, often through a compensation effect, still resulting in an effective transfer coefficient close to 0.5 (Tafel slope of ∼120 mV/dec at 298 K).^13–18^ The large differences in the entropic transfer coefficient, with even the sign changing between liquid and solid mercury electrodes for the same reaction, were attributed to a change in the work function between the solid and liquid metal.^15^

Furthermore, from temperature-dependent studies, the apparent activation energy and pre-exponential factor can be determined as a function of overpotential, which can be extrapolated to zero overpotential to obtain the standard apparent activation energy. Alternatively, the standard apparent activation energy can be determined by the temperature dependence of the exchange current density, with the exchange current density being the extrapolation of the Tafel plot (overpotential vs log *J*) to zero overpotential. In heterogeneous catalysis, as well as for enzymatic catalysis, a compensation effect between the pre-exponential factor and the apparent activation energy is often found, although its origin is not completely understood.^13,19–22^ In a recent study, Sarabia et al. investigated electrochemical hydrogen evolution, ammonia oxidation and oxygen reduction and found consistent trends between the pre-exponential factor and the apparent activation energy for these three reactions,^23^ which were related to solvation effects.

Specifically for the oxygen evolution reaction, temperature-dependent measurements on single-site cobalt catalysts have shown a linear decrease in activation energy with overpotential with an apparent activation energy of ∼37 to 29 kJ/mol between 0.35 and 0.40 V overpotential.^24^ Additionally, CoOx and CoPi catalysts for OER were compared with the enzymatic OER catalyst (photosystem II).^25,26^ The entropy of activation was high for the cobalt-based catalysts, whereas photosystem II had a low entropic contribution to the activation energy. Therefore, it was proposed that reducing the entropy of activation would be a good strategy for improving the activity of heterogeneous OER catalysts. In addition to cobalt-based catalysts, NiFeOOH has been extensively studied for its relatively high oxygen evolution activity and in situ formation from nickel-based catalysts in iron-containing electrolytes.^27–31^ For NiOOH, an activation energy of 72 kJ/mol at zero overpotential has been reported, using the temperature dependence of the exchange current densities, which reduces to ∼25 kJ/mol at optimal Fe loadings (20–30% Fe).^32^ Another study found an activation energy of 25 ± 12 kJ/mol for NiFe on Ni foam (NiFe/NF) also using temperature-dependent exchange current densities.^33^ However, they do not report the corresponding transfer coefficients, temperature-dependent Tafel slopes, or overpotential dependence of the activation energy. Moreover, there is potentially a large contribution of nonkinetic effects, as our previous work has shown by a Tafel slope analysis.^5^ In addition to Tafel slope determination, we expect that nonkinetic effects could also result in large differences in the determined apparent activation energies. Thus, detailed insights into these kinetic parameters are still lacking for the oxygen evolution reaction on NiFeOOH.

Here, we performed OER on NiFeOOH at elevated temperatures in a three-electrode cell with a rotating disk electrode (RDE) under high mass transport conditions, using a low catalyst loading in a moderately concentrated alkaline electrolyte of 0.2 M KOH. We show that, under these conditions, the Tafel slope increases linearly between ∼29 – 36 mV/dec with increasing temperature between 293.15 – 333.15 K, with a large enthalpic transfer coefficient and relatively small and negative entropic transfer coefficient. Interpreting the 30 mV/dec Tafel slope at room temperature would give a mechanism with two electrochemical presteps prior to a chemical RDS or a single electrochemical prestep with a bimolecular chemical RDS. The apparent activation energy was determined at the relevant OER overpotential and was rather small, decreasing from ∼21 to 13 kJ/mol between 0.24 – 0.265 V overpotential. When extrapolating to zero overpotential or by the temperature dependence of the exchange current density, a standard apparent activation energy of ∼75 kJ/mol was obtained, which contains contributions of both the electrochemical presteps as well as the activation energy of the RDS. Interestingly, for a system with higher nonkinetic limitations (4x higher loading, 4x lower KOH concentration), the apparent Tafel slope decreased with increasing temperature, with an inverse trend between the apparent activation energy and the pre-exponential factor with the overpotential. However, when mitigating nonkinetic effects, a kinetic picture consistent with the Butler–Volmer equation was obtained. Our research shows that determining the temperature-dependent kinetic parameters is a valuable approach for obtaining important mechanistic insights into electrocatalysis, but care must be taken in removing nonkinetic limitations.

To determine the temperature dependence of the OER, while ensuring the mitigation of nonkinetic limitations, a low-loading electrodeposited NiFeOOH catalyst (−1.4 mA, 5s),^6^ high rotation rates and a moderately high concentration of 0.2 M KOH were used with 100% *iR* correction.^4^ To suppress the Ni redox contribution to the current, Ni was preoxidized using a CV – CA – LSV procedure, as described previously.^5^ Tafel slope plots were constructed from LSVs at a low scan rate of 1 mV/s, where the Tafel slopes were obtained over small potential regions (5 mV) and plotted vs the average current density or potential. As discussed previously, a horizontal region in a Tafel slope plot is an indication of a fundamental Tafel slope value.^4,5^ Furthermore, it is essential that the real overpotential is used when comparing the temperature-dependent activity, as the thermodynamic equilibrium potential for the OER decreases with temperature. A reversible hydrogen electrode (RHE) was used in this study. Therefore, by definition, the RHE always has HER/HOR equilibrium on Pt at 0 V vs RHE for every temperature, but the OER equilibrium potential will change in the same way as the cell potential for water splitting (values given in Table S1). The overpotential for the OER is then determined as^35^

2 $$\eta_{OER}=E_{RHE}-E_{\left(T\right)}^{0}-0\;V$$

with *η*_*OER*_ the overpotential for the OER, *E*_*RHE*_ the measured potential vs RHE and *E* ^*0*^_*(T)*_ the thermodynamic cell potential of water splitting at the specific temperature, which is the same value as the OER half-cell potential as the HER half-cell potential is 0 V vs RHE.

Figure 1A shows the LSVs (on the overpotential scale) obtained at different temperatures, showing an increase in activity with increasing temperature, as expected. Figure 1B shows the Tafel slope plot at different temperatures (293.15 – 333.15 K), which shows horizontal Tafel slope regions for the different temperatures. Importantly, higher temperatures resulted in higher Tafel slope values, with the Tafel slope values showing a linear trend with the temperature in this temperature range (Figure 1C). This follows the expected trend for a fundamental Tafel slope value, with a transfer coefficient that does not significantly change with temperature (eq 1).^18^

**Figure 1:**
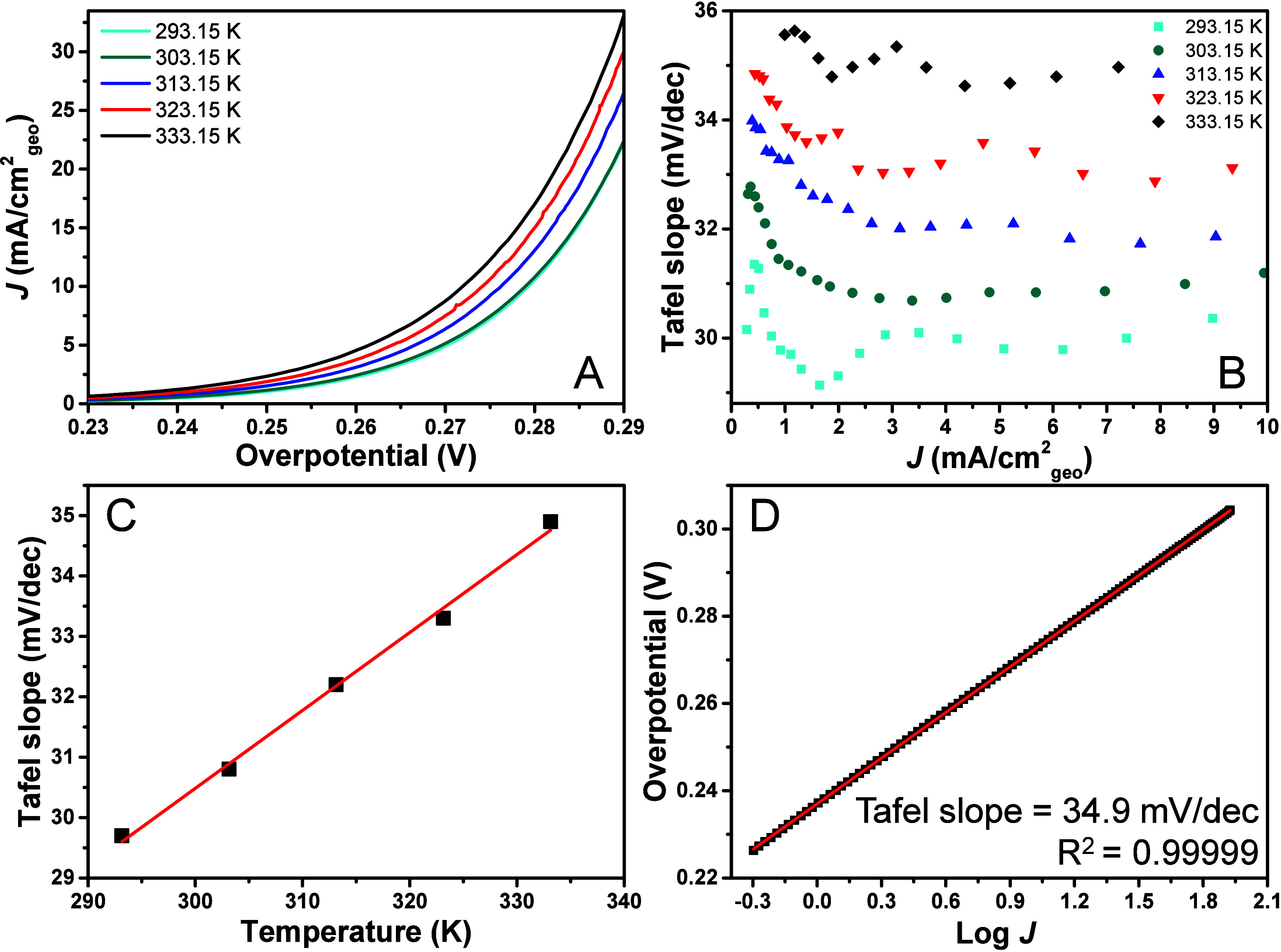
(A) LSVs of NiFeOOH between 293.15 – 333.15 K on an RDE setup at a scan rate of 1 mV/s with 85% *iR* correction in situ and 15% manually afterward, (B) corresponding Tafel slope plots between 0 and 10 mA/cm^2^_geo_, (C) Tafel slope value vs the absolute temperature, as obtained from the horizontal region of the Tafel slope plot in B, (D) “classic” Tafel plot with extended constant Tafel slope for 333 K. To check if the observed changes in Tafel slope value were not due to time effects, newly deposited catalysts were prepared and tested at 273.15 – 303.15 – 333.15 K and showed similar behavior, as shown in Figure S2.

With increasing temperature, the horizontal Tafel slope regions, that is, the potential window with nonchanging Tafel slope values, become more extended. In Figure S1, we show extended Tafel slope plots for different temperatures vs potential, showing that the (apparent) Tafel slope starts increasing earlier at lower temperatures. Figure 1D shows a “classic” Tafel plot at 333.15 K with a linear relationship being observed between log *J* = −0.3 – 1.9. A nonchanging Tafel slope over such a large current region is not often observed for OER catalysts. Therefore, it could be an excellent strategy to perform Tafel analysis at elevated temperatures, since such horizontal regions are apparently much more extended and it would be easier to find fundamental values even for systems that are limited by nonkinetic effects at room temperature. A changing Tafel slope at increasing current density could be explained by the onset of nonkinetic effects, although changes in surface coverage would also result in a changing Tafel slope value. The latter case would typically lead to a second Tafel slope if a wide enough potential window can be measured. However, a second horizontal Tafel slope region, as for example reported for IrOx,^36^ was not observed here for NiFeOOH. Additionally, even if changes in the material as a function of temperature occur, we conclude that their effects on the Tafel slope are minimal.

The observed linear temperature dependence of the Tafel slope provides further evidence that the Tafel slope of ∼30 mV/dec at 298 K is kinetically meaningful and can indeed be regarded as a fundamental value. Using the quasi-equilibrium assumption (which may not always apply^37^) the following equation for the transfer coefficient of the forward reaction is obtained:^38,39^

3 $$\alpha =\frac{n_{b}}{v}+\beta n_{d}$$

with α the effective transfer coefficient, *n*_*b*_ is the number of electrons transferred before the rate-determining step, *v* the occurrences of the rate-determining step in the reaction mechanism (typically 1, but can be 2 as well), β the symmetry coefficient of the rate-determining step and *n*_*d*_ the number of electrons transferred during the rate-determining step. Since a reaction that transfers more than one electron in a single rate-determining step is highly unlikely,^3,40^ *n*_*d*_ takes the value of 1 for an electrochemical step or the value of 0 for a chemical step.

Combining eq 3 with eq 1 gives the Tafel slope value as an indicator of a certain rate-determining step. A Tafel slope of 30 mV/dec is obtained when in the rate-determining step zero electrons are transferred (i.e., a chemical step), with two electrochemical presteps (EEC), giving $59.2\;\frac{\mathrm{mV}}{\mathrm{dec}}/(2+0.5\times 0)$ = 29.6 mV/dec Tafel slope at 298.15 K. Alternatively, a single-electron transfer pre-equilibrium followed by second-order bimolecular reaction (E^2^C) gives the same Tafel slope. A different β, which is often assumed to be 0.5, could also result in different Tafel slope values.^3^ However, for low Tafel slopes this effect is quite small and the only reasonable explanation of a 30 mV/dec Tafel slope is either two electrochemical steps before a chemical RDS, or a single electrochemical step before a bimolecular chemical RDS.^38,41^ In principle, one could consider also a potential-dependent chemical RDS, but given the cardinal value of 30 mV/dec obtained and the lack of direct evidence for strongly potential-dependent chemical steps, we adhere to the traditional interpretation of the 30 mV/dec. Examples of plausible mechanisms are given in Figure S3.

To obtain additional mechanistic information, based on the temperature dependence of the Tafel slope, a so-called Conway plot can be constructed. The Conway plot is the inverse of the Tafel slope vs the inverse of the temperature, as follows:

4 $$\frac{1}{b}=\frac{\alpha_{H}F}{R}\times \frac{1}{T}+\frac{\alpha_{S}F}{R}$$

with *b* the Tafel slope, *F* the Faraday constant, *R* the universal gas constant, *T* the absolute temperature, *α*_*H*_ the enthalpic transfer coefficient and *α*_*S*_ the entropic transfer coefficient. The entropic and enthalpic components of the transfer coefficient can be combined to the overall effective transfer coefficient:^17^

5 $$\alpha_{eff}=\alpha_{H}+T\alpha_{S}$$

Figure 2 shows the Conway plot with data for three separate measurement series, which also shows the spread of Tafel slopes observed in different measurements. From these data, a relatively large enthalpic contribution was determined with *α*_*H*_ = 2.25 ± 0.11, while the entropic component is negative with a value of *Tα*_*S*_ = −0.32 ± 0.10 (at 298.15 K). Therefore, the transfer coefficient and effective activation energy are dominated by the enthalpic contribution, while the entropic contribution is negative and relatively small. This means that the entropy of activation decreases with increasing potential.

**Figure 2:**
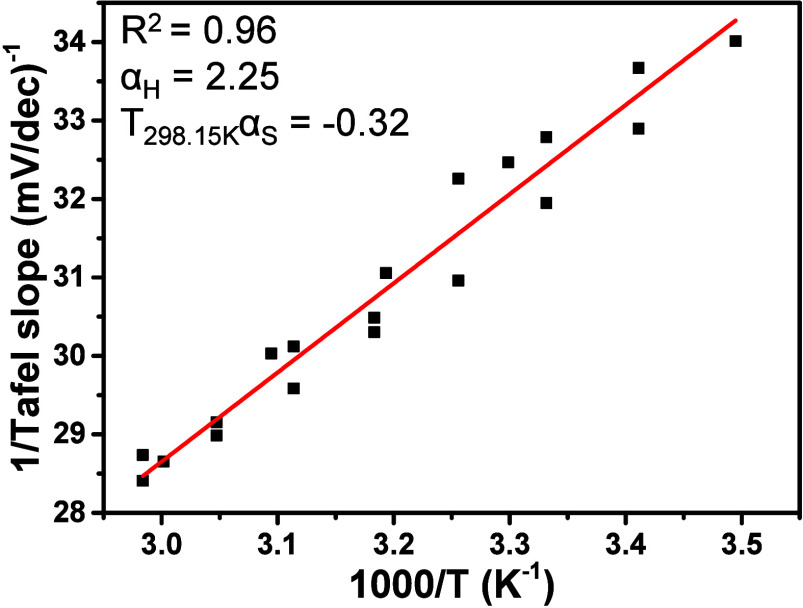
Conway plot of the inverse of the Tafel slope vs the inverse of the temperature. The temperature-dependent Tafel slopes were measured during three experimental series and combined into a single plot; all separate Tafel slope plots are shown in Figure S4.

A similar analysis has been applied previously to amorphous and crystalline iridium oxides (IrOx), with the entropic transfer coefficient determined from the overpotential dependence of the pre-exponential factor. This analysis showed an increasing pre-exponential factor with increasing overpotential for crystalline IrOx and a decreasing pre-exponential factor with increasing overpotential for amorphous IrOx,^35^ corresponding to an increasing and a decreasing entropy of activation with potential, respectively. The low pre-exponential factor is at the root of the lower rate of crystalline IrOx toward the OER, even though the observed apparent activation energy for crystalline IrOx is lower, at least in a certain potential window. However, the real meaning of such conclusions remains uncertain, as there is a clear but largely unresolved compensation effect. We note that for interfacial electrochemical reactions, Conway considers a potential-dependent interfacial solvent structure as the main cause for a potential-dependent entropic contribution to the activation energy, and hence to a sizable *Tα*_*S*_.^12^ This would mean that the negative entropic transfer coefficient, as observed here, could be explained as a slightly more favorable interfacial solvent structure with increasing potential.^12^

From the temperature-dependent measurements, the standard apparent activation energy was determined using two methods: either by plotting the natural log of the current vs 1/*T* at different overpotentials (in the horizontal Tafel slope region between 0.240 and 0.265 V overpotential, as shown in Figure S1), which is then extrapolated to zero overpotential, or from the temperature dependence of the exchange current density (extrapolation of the Tafel plot to zero overpotential). The potential window of 25 mV to determine the kinetic parameters is relatively small, but unavoidable due to the onset of nonkinetic effects. When interpreting the Arrhenius parameters, the apparent activation energy relates to the enthalpy of activation, whereas the pre-exponential factor relates to the entropy of activation, which together determine the Gibbs free energy of activation.^12^ For clarity, only one measurement is shown in Figure 3, whereas the results from other measurements are tabulated in Table 1 and are shown in Figure S4. Figure 3A shows the exchange current density vs 1/*T*, resulting in a standard apparent apparent activation energy of 75.5 ± 8.4 kJ/mol when averaged over the 3 measurements. Figure 3B shows the apparent activation energy vs the overpotential between 0.240 and 0.265 (horizontal Tafel slope region) as determined from the temperature-dependent current density at the same overpotential showing a linear decrease in apparent activation energy with increasing overpotential. A standard apparent activation energy of 75.3 ± 2.1 kJ/mol was then determined by extrapolation to zero overpotential and from the slope the enthalpic transfer coefficient of 2.40 ± 0.03 is obtained. Figure 3C shows the pre-exponential factor as a function of overpotential, from which a negative entropic transfer coefficient of −0.50 ± 0.03 was determined (at room temperature). Table 1 shows that the determined kinetic parameters are quite consistent, also with the Conway plot (Figure 2), and result in similar Tafel slopes when the transfer coefficients are reinserted in eq 1. This is not surprising, as the same data are at the base of these extrapolations, however inconsistencies can arise when the potential dependent activation energies are determined over a different potential region than the exchange current densities and the Tafel slope plot does not show a horizontal region.

**Figure 3:**
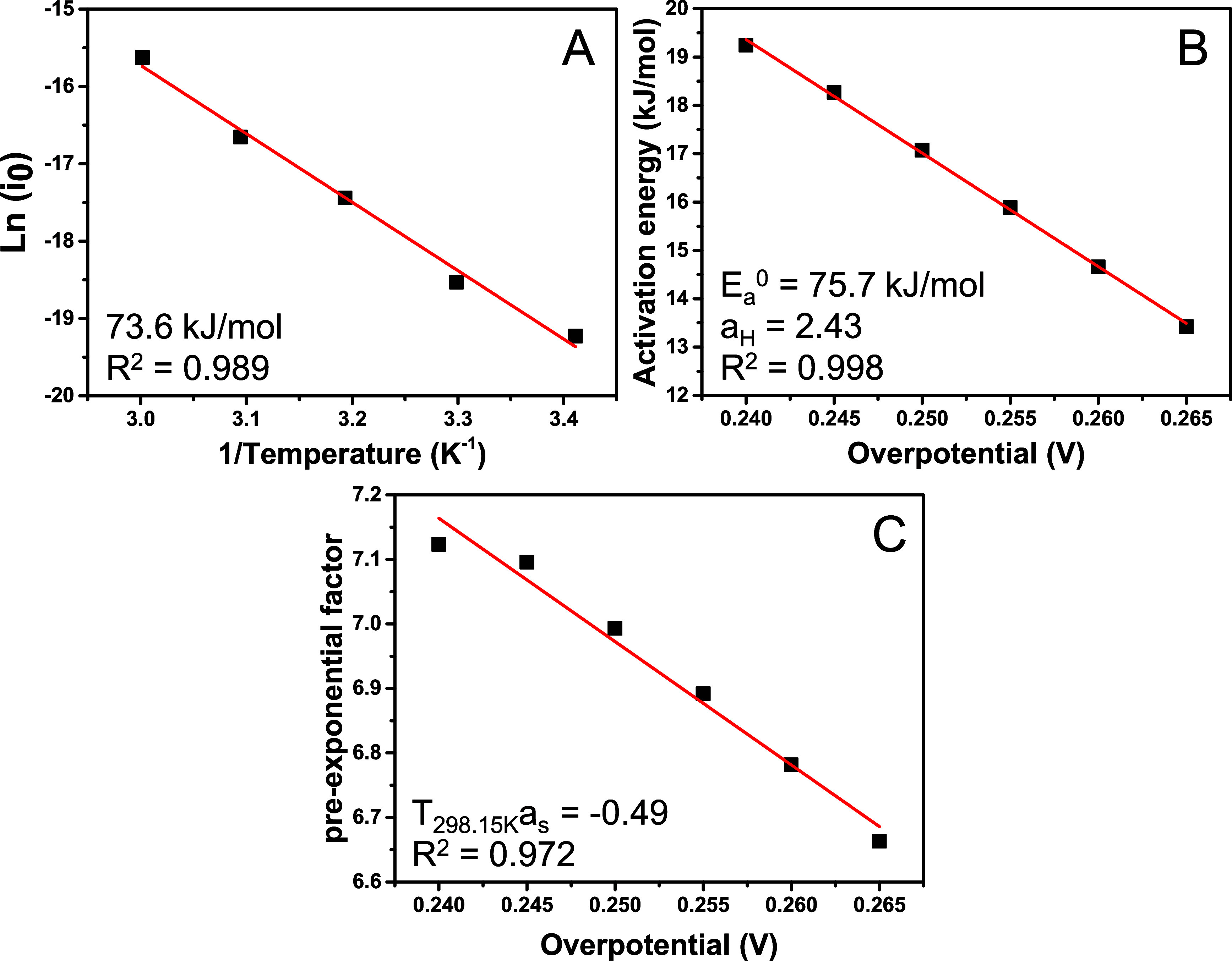
(A) Arrhenius plot of the temperature-dependent standard exchange current density, giving the standard apparent activation energy, (B) apparent activation energy vs overpotential, yielding a standard apparent activation energy (i.e., at zero overpotential) of 75.7 kJ/mol, (C) pre-exponential factor vs the overpotential between 0.240 and 0.265 V. The overpotential dependence of both the activation energy and pre-exponential factor over a larger potential window is shown in Figure S4.

**Table 1:** Overview of Standard Activation Energies, Enthalpic and Entropic Transfer Coefficients, and Tafel Slopes Calculated from *b* = *RT*/α*F* with α = α*_H_* + *Tα_S_* and *T* = 298.15 K

	*E*^0^_*act*_ (from *E*_*a*_ vs overpotential)	*E*^0^_*act*_ (from *T*-dependent *i*_0_)	α_H_	*T*_(298.15K)_α_*S*_	Calculated Tafel slope (mV/dec)
**1**	75.7	73.6	2.43	–0.49	30.0
**2**	77.1	84.7	2.40	–0.47	30.2
**3**	72.9	68.2	2.37	–0.54	31.7
**Average (1–3)**	75.3 ± 2.1	75.5 ± 8.4	2.40 ± 0.03	–0.50 ± 0.03	30.6 ± 1.0
**Conway plot (1–3)**	-	-	2.25 ± 0.11	–0.32 ± 0.10	30.0

If the linear trend of Figure 3B would continue, then further increasing the overpotential would result in a negative apparent activation energy at a sufficiently high overpotential. However, Figure S4 shows the apparent activation energy over a wider potential region, where the apparent activation energy starts to level off, with corresponding changes in both the Tafel slope and the pre-exponential factor. Unfortunately, the different measurements are not perfectly consistent, and it is difficult to exclude nonkinetic effects at these relatively high current densities. AC voltammetry, or spectroscopy, could provide more insight into whether the Tafel slope change is caused by changes in the surface coverage by determining the *E* ^0^ of the pre-equilibrium step.^42^ If this occurs, the surface coverage of the initial species in the RDS can no longer be assumed to be small, thus resulting in a change in the Tafel slope.

It is important to note that the interpretation of the apparent activation energy depends on the mechanism and is therefore different for different fundamental Tafel slope values. In the case of pre-equilibrium steps, the (apparent) activation energy also contains the (potential-dependent) reaction energy of those presteps.^25^ As discussed above, the rate-determining step is a chemical step preceded by two electrochemical equilibrium steps or a single electrochemical equilibrium step followed by a bimolecular chemical RDS. Therefore, the measured potential dependence of the activation energy cannot be explained by the chemical RDS, but should be attributed to the potential dependence of the free energies of the electrochemical presteps. This also means that the activation energy of the chemical step is only, at maximum, the lowest activation energy found, which is ∼13 kJ/mol at 0.265 V, or if the potential window is extended, ∼8 kJ/mol (Figure S4). A schematic of the energy diagram is shown in Figure 4, with the standard apparent activation energy containing the free energy of both presteps, as well as the activation energy of the rate-determining step. The potential-dependent apparent activation energy therefore also includes the potential-dependent binding energies of the intermediates.

**Figure 4:**
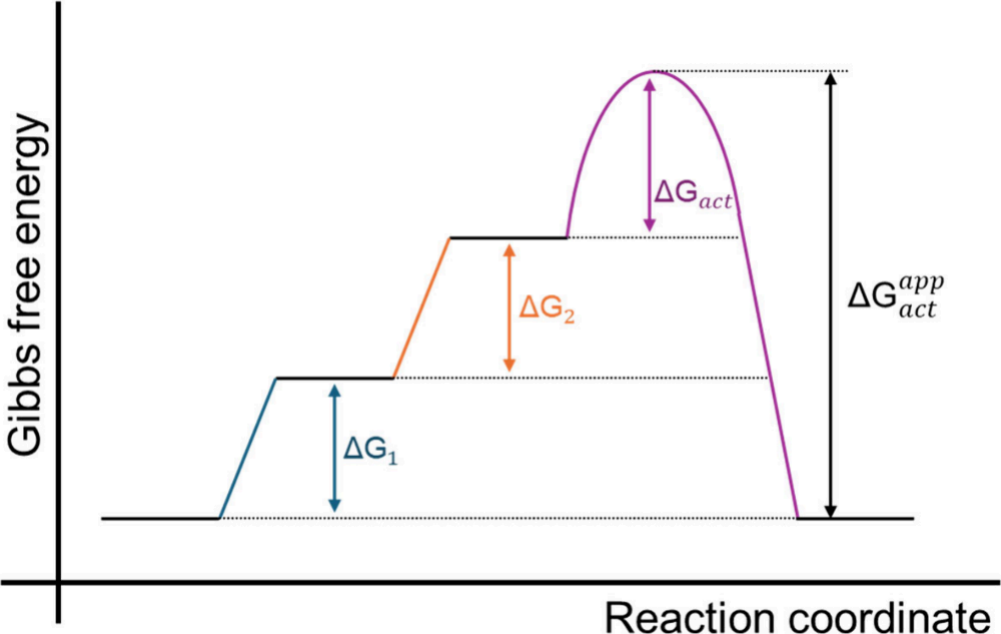
Schematic energy diagram of a mechanism with two electron transfers before the chemical rate-determining step and different contributions to the standard apparent activation energy (drawn at zero overpotential).

Importantly, it is essential to mitigate nonkinetic effects when measuring the kinetic parameters of electrocatalytic reactions. To demonstrate how a significant contribution of such nonkinetic effects changes the apparent activation parameters, experiments were performed at a four times higher loading and four times lower KOH concentration (0.05 M KOH) and lower rotation rate (1500 rpm). These conditions were previously shown to give rise to mass transport limitations, due to more mass transport limitations within the catalyst layer, as well as reduced removal of gas bubbles that increase ohmic resistance and block active sites.^5^ Figure 5A shows the corresponding LSVs. Figure 5B shows the Tafel slope plots under these conditions, where the Tafel slope decreases with increasing temperature, in stark contrast to Figure 1B, and no extended horizontal region is observed. The lowering of the Tafel slope with temperature could indicate a substantial reduction in the nonkinetic limitations with higher temperature. This is consistent with the reduced mass transfer limitations and enhanced conductivity within the catalyst and the electrolyte, among others, because the viscosity of water decreases at higher temperatures.^43^ Therefore, changing the temperature can be a useful tool to assess the significance of nonkinetic contribution to the measured Tafel slope.

**Figure 5:**
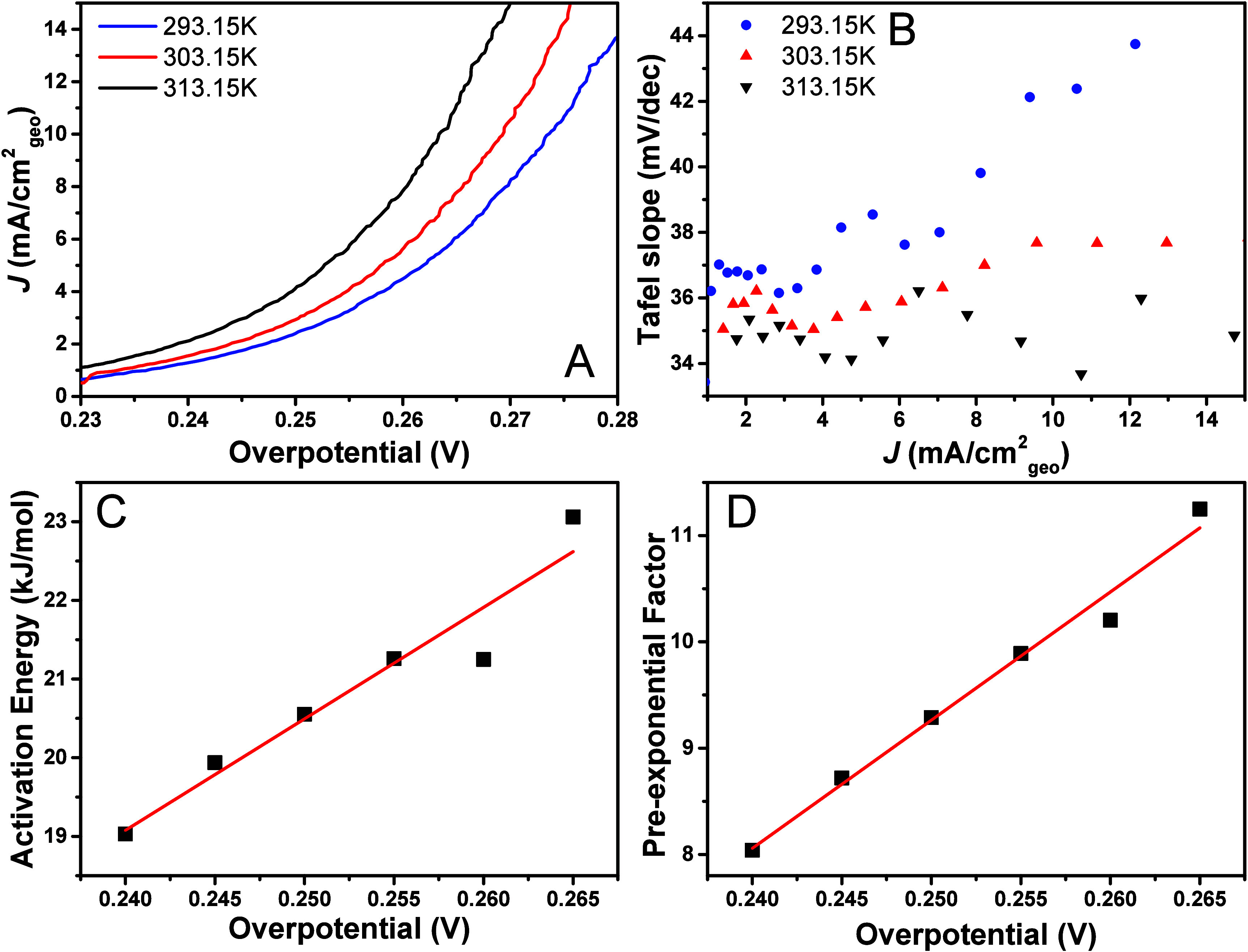
(A) LSVs at 293.15, 303.15, and 313.15 K for a catalyst and electrolyte that is more likely to give rise to nonkinetic limitations: four times larger loading, four times lower KOH concentration, and a lower rotation rate of 1500 rpm, (B) corresponding Tafel slope plots with higher apparent Tafel slope at lower temperatures due to increased contribution of nonkinetic effects, (C) apparent activation energy (293.15 – 313.15 K) as a function of overpotential, (D) pre-exponential factor as a function of overpotential. Note that there is slightly more Ni oxidation contribution to the Tafel slope (B) at low current densities due to the higher loading.

Figure 5C shows the apparent activation energies as a function of potential between 293.15 – 313.15 °C. In line with the changes in apparent Tafel slope, the apparent activation energy increases with overpotential, resulting in a negative standard apparent activation energy when extrapolated to zero overpotential. Although this appears unphysical, recall that the apparent activation energy is in fact an activation enthalpy, and a negative activation enthalpy can still yield a positive free energy of activation if the activation entropy is sufficiently negative.^12^ However, in this case we attribute these counterintuitive trends to nonkinetic contributions to the current. Interestingly, Figure 5D shows that the apparent pre-exponential factor increases with increasing overpotential. This shows the strongly compensating trends between apparent pre-exponential factor and apparent activation energy. One of the hypotheses of the observed compensation effect is that it is merely a result of experimental variation in combination with large extrapolations, which is something to keep in mind when performing measurements that include nonkinetic effects that convolute the kinetic analysis.^21,44^ Another method to provide an indication of the nonkinetic limitations related to internal mass transport in the catalyst layer is to study the reversibility of the Ni reduction peak.^45^ Figure S5 shows that the Ni redox peaks become more reversible at higher temperatures, probably because of facilitated internal mass transport through the catalyst layer. Therefore, if this internal mass transport also has a significant impact on the OER activity, it will convolute the determined kinetic parameters. An important general conclusion is that these nonkinetic effects may be strongly temperature-dependent and may therefore give rise to uncharacteristic and apparently unphysical trends in the apparent activation energy.

In the literature, the activation energy for the OER is either given at a certain overpotential or is extrapolated to the standard equilibrium potential (from the temperature-dependent exchange current density). We believe that it is important to also show the overpotential dependence of the activation energy, which should result in a consistent value for the standard apparent activation energy, but only when determined in a horizontal Tafel slope region in the Tafel slope plot, that is, without any significant nonkinetic limitations. In the literature, often Tafel slopes that decrease with increasing temperature were observed for OER,^26,33,46–48^ although also constant Tafel slope values with temperature were found.^49^ For example, very large reductions in Tafel slopes were found at increased temperatures, e.g. from 93.1 mV/dec at 10 °C, 72.8 mV/dec at 20 °C to 56.2 mV/dec at 30 °C for porous Ni foam.^46^ Such a porous Ni foam is likely to be strongly affected by the internal mass transport in the pores, and such a strong decrease in the Tafel slope will probably not be observed for thinner OER catalyst layers. However, similar contributions, albeit to a smaller extent, will still strongly convolute these measurements, as shown in Figure 5. A decreasing Tafel slope with increasing temperature is not expected from the Butler–Volmer equation unless there is a large temperature dependence of the transfer coefficient. Determining the Tafel slope as a function of temperature, preferably using Tafel slope plots, is a powerful method to ascertain their kinetic meaningfulness. Therefore, intrinsic kinetic insights can only be obtained when eliminating nonkinetic effects, because the interpretation of the standard apparent activation energies (and their potential-dependent behavior) depends on the mechanism and therefore on the fundamental Tafel slope value.

Finally, from the kinetic data presented above, we can consider a more detailed mechanistic interpretation, especially in relation to what has been reported in the literature. When considering the full molecular structure, as done by Wang et al.,^50^ three common mechanisms can be considered: the lattice oxygen mechanism (LOM), adsorbate evolution mechanism (AEM, Figure S3A, C), and intramolecular oxygen coupling (IMOC, Figure S3B). The LOM mechanism is differentiated from the AEM and IMOC by the origin of the oxygen, where the oxygen originates from the lattice for the LOM, from a surface adsorbate and a species from the electrolyte for the AEM, and from the combination of two surface adsorbates for the IMOC. As described earlier, the mechanism should proceed through either two electrochemical presteps followed by a chemical RDS (EEC) or an electrochemical prestep followed by a bimolecular chemical RDS (E^2^C), which is possible for both the IMOC and LOM, but not for the electrochemical AEM. These mechanisms can be differentiated by other methods. For instance, the occurrence of a lattice oxygen-mediated mechanism can be observed by isotope labeling. Multiple studies have shown that the LOM is not dominant for NiFeOOH, whereas it is dominant for NiOOH.^50–54^ Dynamic iron sites have been shown to be the main contributors to the OER activity of NiFeOOH.^30^ The different mechanism for NiOOH in Fe-free solution is also evident from the large difference in the reported Tafel slopes for NiFeOOH.^55^ Therefore, NiOOH would most likely provide distinct temperature-dependent behavior.

Experimentally, it was observed that the Tafel slope decreased toward 30 mV/dec with increasing Fe content in the electrolyte,^28,30^ consistent with the 30 mV/dec Tafel slope of Ni_80_Fe_20_OOH observed here at room temperature for NiFeOOH. Wang et al. calculated, based on DFT, that these dynamic (or “fleeting”) Fe sites mainly follow an IMOC mechanism with a chemical O–O coupling as rate-determining step on an adsorbed Fe site, resulting in a Tafel slope close to 30 mV/dec.^53^ Ou et al. also presented a mechanism on surface adsorbed Fe sites, though in their mechanism this is a surface adsorbed Fe–O–Fe dimer, for which they consider the bridge site to have the lowest overpotential.^28^ Still, the intramolecular oxygen coupling mechanism on either a single or two surface-adsorbed Fe site(s) with chemical oxygen coupling as the RDS is consistent with what has been observed here. Further research could provide further insights by determining the *E* ^0^ of the pre-equilibrium step(s) with e.g. AC-voltammetry^42^ or by detecting relevant intermediates spectroscopically. Nevertheless, temperature-dependent studies allow for important mechanistic insights that could improve the understanding of the OER. Specifically, they allow for improved comparison of intrinsic kinetic properties by comparing fundamental Tafel slope values, transfer coefficients, and potential dependent (standard) apparent activation energies of different relevant OER catalysts, on the condition that they include negligible nonkinetic effects.

In conclusion, the temperature dependence of the oxygen evolution reaction on NiFeOOH in 0.2 M KOH was studied, and a multitude of kinetic parameters was extracted. First, the temperature dependence of the Tafel slope was investigated, and it was found that the Tafel slope increases linearly with temperature (between 293.15 and 333.15 K) in accordance with the Butler–Volmer equation with a Tafel slope of ∼30 mV/dec at 298.15 K. Moreover, Tafel slope plots at higher temperatures show more extended horizontal Tafel slope regions. For example, a consistent Tafel slope value between log *J* = −0.3 – 1.9 at 333.15 K was observed. Such extended linear Tafel regions are not commonly observed for OER electrocatalysts. This indicates that it would be generally beneficial to determine the Tafel slope values at elevated temperatures, where the horizontal Tafel slope regions could be obtained for systems that have significant nonkinetic limitations at room temperature. Moreover, from the Conway plot (1/Tafel slope vs 1/temperature) a large enthalpic transfer coefficient of 2.25 ± 0.11 and a small and negative entropic transfer coefficient of −0.32 ± 0.10 were found. This means that the entropy of activation slightly decreases with an increasing potential.

Furthermore, the apparent activation energy was determined in the potential region where the Tafel slope plots were horizontal between 293.15 and 333.15 K (overpotential of 0.240 – 0.265 V). From the potential dependence of the activation energy with overpotential, a standard apparent activation energy of 75.2 ± 2.1 kJ/mol was determined, with the apparent activation energy decreasing with increasing overpotential. A similar standard apparent activation energy of 75.5 ± 8.4 kJ/mol was extracted from the temperature dependence of the exchange current densities. At a relevant OER overpotential (0.265 V) the apparent activation energy was only ∼13 kJ/mol. Similar transfer coefficients to the Conway plot were observed (2.40 ± 0.03, −0.50 ± 0.03), showing a large enthalpic contribution to the potential dependence of the Gibbs energy of activation with a smaller and negative entropic contribution. Moreover, our analysis demonstrates that it is essential that the kinetic parameters are determined free of nonkinetic effects, as the temperature strongly affects the internal mass transport and possibly the bubble behavior. A decrease in the Tafel slope with temperature was observed for a catalyst with a higher loading and a lower KOH concentration, resulting in an unlikely increase in the apparent activation energy with increasing overpotential combined with a negative standard apparent activation energy (if extrapolated).

Additionally, the linear increase of the Tafel slope with temperature provides an additional verification that the 30 mV/dec Tafel slope at room temperature is fundamental. The 30 mV/dec Tafel slope suggests a mechanism with two equilibrium electrochemical steps prior to a chemical rate-determining step or an equilibrium electrochemical step prior to a bimolecular chemical rate-determining step. Therefore, the value of the Tafel slope is essential for the interpretation of the standard apparent activation energy as the interpretation depends on the mechanism. This means that the standard apparent activation energy measured here mostly consists of the free energy of the (two) electrochemical presteps. The chemical rate-determining step has a rather small activation energy with a relatively small decrease in the entropy of activation with increasing overpotential. An example of such a mechanism could be the O–O coupling between two adsorbed oxygen species on the catalyst surface (IMOC mechanism), following a Nernstian conversion between *OH and *O. However, multiple interpretations could be consistent with the observations presented here, and the identification of the detailed mechanism cannot be based on these measurements alone. Nonetheless, temperature-dependent electrocatalytic measurements, if they mitigate nonkinetic effects, can provide important insights into the operation of electrocatalysts.

## Supplementary Material



## Notes

Experimental details, table of temperature-dependent half-cell potential for OER, additional temperature-dependent experiments, temperature dependence of the reduction peak (PDF)
