# Supplementary material for: Temperature-Dependent Kinetic Parameters for the Alkaline Oxygen Evolution Reaction on NiFeOOH
Source: ACS Energy Lett. 2025 Jun 3;10(6):3040–9. doi: 10.1021/acsenergylett.5c01387 (PMC12172033; doi:10.1021/acsenergylett.5c01387)
Supplement: Supplementary file 1 [file nz5c01387_si_001.pdf]

Supporting information to:

Temperature-Dependent Kinetic Parameters for the Alkaline Oxygen Evolution Reaction on NiFeOOH

Onno van der Heijden, Rafaël E. Vos and Marc T.M. Koper

Leiden Institute of Chemistry, Leiden University, 2333 CC, Leiden, the Netherlands.

Contents

Experimental........2

Thermodynamic equilibrium potential at different temperatures........5

Tafel slope plot of Figure 1 vs. potential........6

Tafel slope at different temperatures for newly deposited catalysts........7

Plausible mechanisms........8

Activation energy and pre-exponential factor over a larger potential range........10

Ni redox peaks........11

References........12

Experimental

General cleaning procedures

Glassware and plastic cells were stored in 0.5 M H_2SO_4 (95-98%, ACS reagent, Sigma-Aldrich) solution containing 1 g/L KMnO4 (>99%, ACS reagent, Emsure). Before use, the glassware was cleaned with a diluted piranha solution (H_2O_2, 35%, Merck and H_2SO_4, 95-98%, ACS reagent, Sigma-Aldrich) and boiled in Milli-Q water (resistance: 18.2 MΩ.cm) at least three times.

Oxygen evolution reaction, RDE experiments, heating bath

A rotating disk electrode (RDE) setup was used (MSR Rotator, Pine Research). The substrate for the working electrode was a fixed gold disk in a PEEK sheath in a shaft for increased rotation rate, which does not contain a Teflon cup (E2MPK FastSpeed RDE, gold disk: 5 mm OD, 0.196 cm^2, Pine Research). First, the gold disk was polished on a microcloth (Buehler) with diamond suspension of 3, 1, 0.25 and 0.05 µm (MetaDi), respectively. Thereafter, the tip was sonicated for at least 10 min to remove attached diamond particles.

Prior to the electrodeposition of the catalyst, the gold substrate was cycled in a three-electrode cell with the RDE as the working electrode, a platinum counter electrode, and a Hydroflex® (Gaskatel) reversible hydrogen reference electrode. To check that the substrate does not leak and is stable, CV scans were conducted in the range 0.05 – 1.75 V vs. RHE at 50 mV/s in 0.1 M H_2SO_4 (96%, Suprapur, Merck), for which it is important to check that the Au reduction peak is stable and does not continuously increase with cycling. To deposit the NiFe catalyst precursor, a method similar to that of Chakthranont et al. was used[1]. The following cathodic deposition conditions were used: -1.4 mA for 5 s with a constant rotation rate of 400 RPM in a 80 mM $Ni(NO_3)_2.6H_2O$ (99.99% trace metal basis, Sigma-Aldrich) + 20 mM $FeSO_4.7H_2O$ (>99%, ACS reagent, Sigma-Aldrich) solution in milliQ water. The deposition electrolyte was prepared from a 80 mM $Ni(NO_3)_2.6H_2O$ stock solution to which $FeSO_4.7H_2O$ salt was added after purging the $Ni(NO_3)_2$ solution with argon for at least 15 minutes to prevent FeOx formation. The deposition procedure was previously followed by eQCM, the resulting layer after electrodeposition has been characterised using SEM and the Ni and Fe ratio has been confirmed by ICP-MS in a previous study reported elsewhere[2].

For the oxygen evolution reaction, a three-electrode setup with a rotator was used, with the deposited NiFe layer on the Au disk as described above using a water bath (Lauda, Ecoline Staredition E100) to control the temperature. To prevent glass dissolution into the electrolyte, a plastic (Nalgene®) cell was used[3], a large surface area gold wire (99%, 0.8 mm thick, Mateck) was used as the counter electrode, and a Hydroflex® RHE electrode (Gaskatel) was used as the reference electrode. The use of a certain reference electrode is important for studying temperature-dependent electrocatalytic

reactions. The advantage of using the RHE at the same temperature as the cell is that the equilibrium potential of the HER/HOR on Pt is 0 V vs. RHE_T[4]. The equilibrium potential of the OER is then given by the cell potential for water splitting E^0 – 0 V, which means that the overpotential is given by the measured potential minus the equilibrium potential of the OER at that temperature; these values are given in Supplementary Table 1.

A 1 M stock solution was prepared from KOH pellets (99.99% semiconductor grade, 15% water, Sigma-Aldrich), the resulting solution was made iron free with the method described before[5]. The iron was scavenged by dispersed $Ni(OH)_2$ in the 1 M KOH electrolyte in a centrifugal tube, next the electrolyte was prepared by centrifuging at 6000 RPM for 10 min, after which the required amount of electrolyte was transferred by a pipet to the plastic cell containing miliQ water, resulting in a 0.2 M KOH solution. However, some iron contamination in the final electrolyte cannot be ruled out, as the catalyst contains 20% iron. Prior to the reported measurements the catalyst layer was activated by at least 50 scans at 50 mV/s (different potential ranges depending on the temperature) with the potential scanned into the OER region towards (slightly) more anodic than to the potential towards which the (CV-CA)-LSV measurement of interest was performed.

Prior to the measurement for the determination of the kinetic parameters, it was verified that the temperature of the electrolyte was constant by measuring repeated impedance spectra at the open circuit potential (OCP). The solution resistance decreased during heating and then became stable again when the temperatures of the heating bath and electrolyte were similar. Then, at least five CVs cycles, similar to the activation procedure, were run to ensure that the catalyst was stable. The CV-CA-LSV procedure, similar to what was introduced previously[6], was performed to remove the majority of the Ni oxidation contribution at low current densities. The CV was taken at 10 mV/s from 1.2 V vs. RHE to OER potentials and back to just before Ni reduction starts, after which a CA was performed for 10 s at that same potential. Note that these potentials on the RHE scale are slightly different for each temperature. Finally, an LSV was taken, in which the potential is swept over the relevant potential region for OER for the different temperatures, starting just after Ni oxidation towards at least 30 mA/cm^2_{geo} of current. The LSV was usually performed at a scan rate of 1 mV/s, or another scan rate when specified. The rotation rate was maintained at a rate that would provide similar mass transport conditions with regard to the Reynolds number, meaning that the rotation rate was lowered with increasing temperature owing to reduced viscosity at elevated temperatures[7], following[8]:

$$Re = \frac{\omega r^2}{\nu} \tag{1}$$

with ω the angular velocity, r the radius of the electrode and v the kinematic viscosity. The rotation rate was set to 4000 RPM at 293.15 K, 3000 RPM at 303.15 K, 2500 RPM at 313.15 K, 2000 RPM at 323.15 K and 1800 RPM at 333.15 K or intermediate value for an intermediate temperature. For experiments with high non-kinetic effects, the rotation rate was maintained at 1500 RPM. The ohmic resistance was determined from impedance measurement and compensated for 85% in situ and for 15% manually afterwards. Impedance spectroscopy (50 kHz – 100 Hz, 10 mV amplitude) at multiple OER potentials (at low currents) was performed to determine this ohmic resistance during OER. Moreover, the ohmic resistance was determined both before and after the CV-CA-LSV procedure to check whether the ohmic resistance had not changed significantly during the measurement. The Tafel slope plots[6,9] were then determined from these LSVs over 5 mV intervals. Moreover, a so-called Conway plot was determined, i.e. inverse Tafel slope vs. inverse temperature, where the Tafel slope was taken from the horizontal region in the Tafel slope plot for each temperature. A single Tafel slope value was determined from the LSV in a "traditional" log J vs. E plot over the horizontal region in the Tafel slope plot with a range of at least 20 mV, but larger at higher temperature. The enthalpic transfer coefficient was determined from the slope of the Conway plot and the entropic transfer coefficient from the intercept.

The Arrhenius parameters were determined from these LSVs at the same overpotential from the plot of the natural log of the current vs. the inverse of the temperature. The apparent activation energy is determined from the slope and the pre-exponential factor from the intersect of this plot. Both were determined as a function of overpotential, whereby the standard apparent activation energy is determined by the extrapolation of the apparent activation energy to zero overpotential. The potential dependence of the activation energy also gives access to the enthalpic transfer coefficient and the potential dependence of the pre-exponential factor gives access to the entropic transfer coefficient. Moreover, the exchange current density was determined from the extrapolation of the Tafel plot (in the horizontal region of the Tafel slope plot) to zero overpotential at the different temperatures. The standard apparent activation energy can then be determined from the corresponding Arrhenius plot. The error bars were determined as the standard deviation from three separate measurements

Thermodynamic equilibrium potential at different temperatures

Thermodynamically, the equillibrium potential can be determined from the tabulated ΔH and ΔS values, in this case taken from the NIST data base[10] and the T the temperature of interest.

$$\Delta G = \Delta H - T\Delta S \quad \text{(S1)}$$

with ΔG the Gibbs free energy of formation, ΔH the enthalpy of formation and ΔS the entropy of formation. With the obtained ΔG value, the cell equillibrium potential can be determined as follows:

$$E^0 = -\Delta G/nF \quad \text{(S2)}$$

with E^0 the equillibrium potential of the cell, n the number of electrons transferred and F Faraday constant. As the potential of the hydrogen equillibrium potential at the RHE scale is by definition 0 at all pH values, the following redox potentials are also the equillibirum potential of the OER on the RHE scale, as given in Table S1.

Table S1. Equilibrium half-cell potentials of the oxygen evolution reaction to the absolute temperature.

**T (K)**	**E^0 (V vs. RHE)**
293	1.234
303	1.225
313	1.216
323	1.208
333	1.199

Tafel slope plot of Figure 1 vs. potential

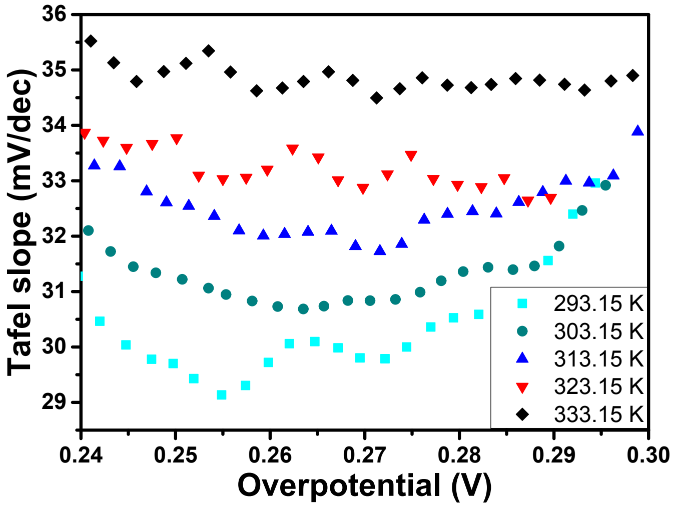

Figure S1. Tafel slope plots vs. the overpotential determined at different temperatures, from the LSVs of Figure 1 in the main text, showing more extended horizontal Tafel slope regions at higher temperature.

Tafel slope at different temperatures for newly deposited catalysts

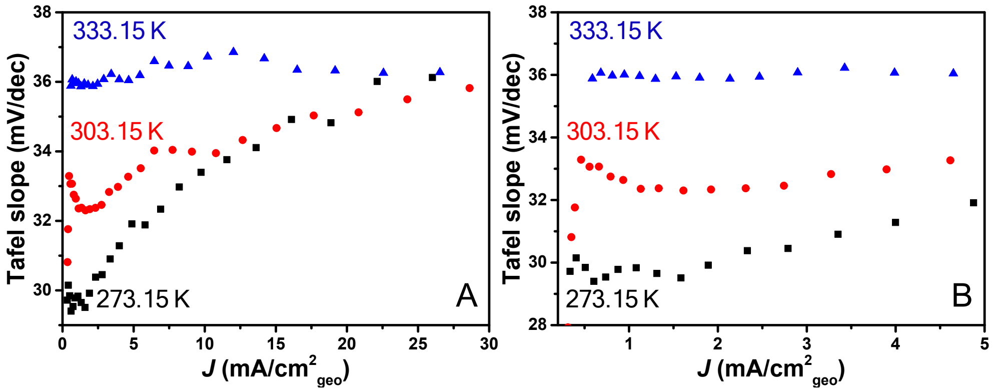

Figure S2. (A) Tafel slope plot of newly deposited catalyst layer at 273.15, 303.15 and 333.15 K, (B) zoomed in on the low current density. An increase in Tafel slope at higher temperatures was observed, in accordance with Figure 1 of the main text. Moreover, the same behaviour with increasing current density was observed.

Plausible mechanisms

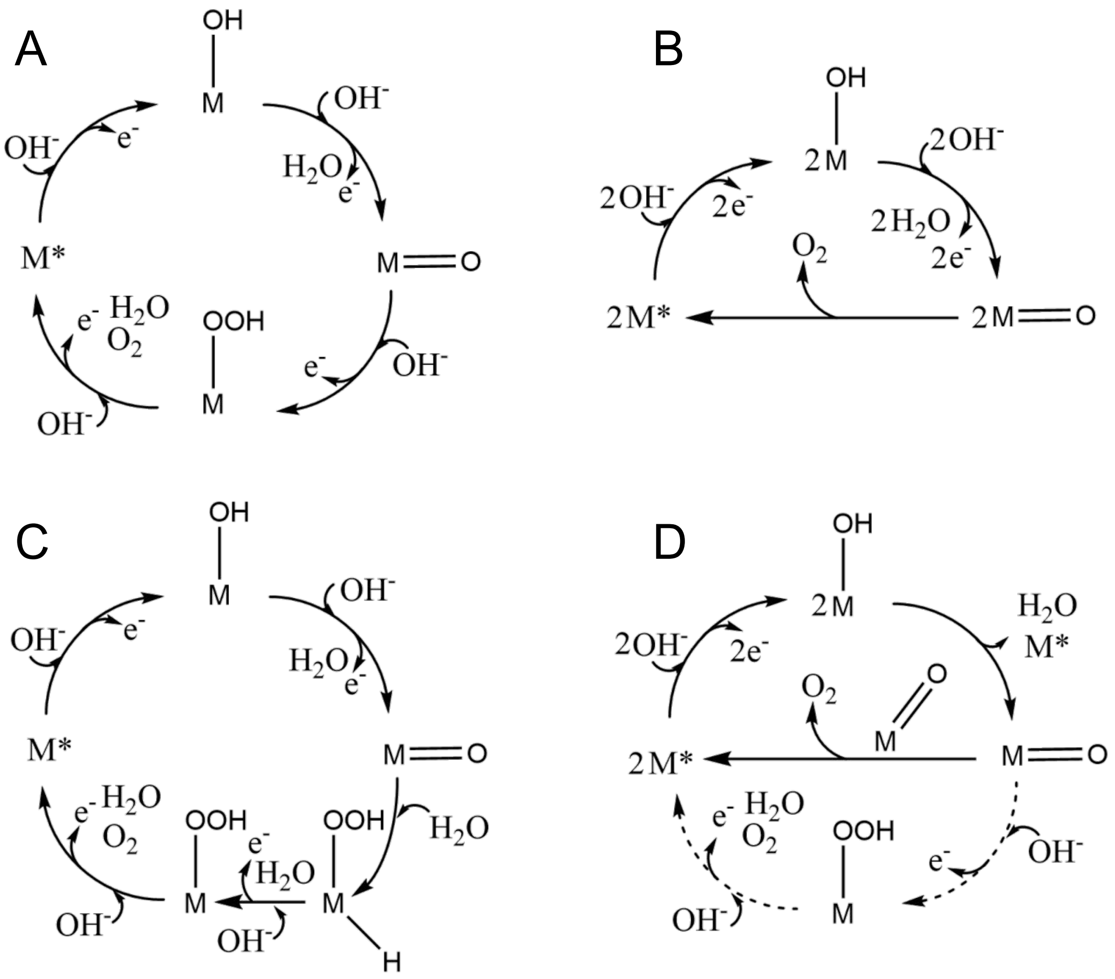

Figure S3. Four possible OER mechanisms[11,12]: (A) adsorbate evolution mechanism (AEM) single-site mechanism with four proton-coupled electron transfers (PCET), (B) dual-site mechanism with chemical O-O coupling of two M=O, (C) AEM single-site mechanism with a chemical O-O coupling involving a water molecule from the electrolyte and (D) mechanism with disproportionation of M-OH to M=O with subsequent M=O coupling to form oxygen (top), or alternatively the same path from M=O can be followed as in A (dotted lines).

To combine the above-mentioned insight with possible OER mechanisms, four plausible mechanisms are given in Figure 2. Figure 2A shows the commonly used single-site electrochemical adsorbate mechanism. However, the observed Tafel slope of 30 mV/dec excludes this commonly reported OER mechanism for NiFeOOH, as it only contains electrochemical steps and can therefore not explain a Tafel slope of 30 mV/dec. Figure 2B shows a dual-site mechanism that follows two similar electrochemical steps to obtain two surface-adsorbed M=O, which then undergo chemical coupling of oxygen. If this chemical oxygen coupling is rate-determining with the M=O formed in an electrochemical equilibrium (starting the catalytic cycle from MOH, i.e. the MOH coverage is close to 1), this would give a Tafel slope of 30 mV/dec and is therefore a possible (E^2C) mechanism in

agreement with the observed Tafel slope. Important to note is that it is essential to interpret the correct resting point of the catalytic cycle, which is based on the surface coverage and therefore can change with potential. More detailed information can be obtained when applying micro-kinetic modelling using the steady state assumption instead of the quasi equilibrium assumption[13–15]. Still, multiple different options can be considered, which are in agreement with the Tafel slope of 30 mV/dec. For example, one could consider the same two electrochemical steps as the AEM in (A), but with a chemical oxygen coupling through a water from the electrolyte, as shown in Figure 2C. This would give a chemical rate-determining step after two electrochemical steps (EEC), in agreement with the 30 mV/dec Tafel slope. Furthermore, the disproportionation of two MOH species, as shown in Figure 2D, is another interesting option to envisage a mechanism with a possible chemical rate-determining step. In this mechanism electrochemical steps result in the formation of M-OH, while the subsequent reaction steps can be either chemical (top) or electrochemical (bottom half cycle, similar to A). Besides the four mechanism shown in Figure 2, other plausible mechanism could also be envisaged[16]. Although a specific Tafel slope does limit the number of possible mechanisms and provides information on the nature of the rate-determining step, it does not indicate a unique mechanism. Also note that the mechanism drawn in Figure 2 assumes a single type of active site with a single functionality. For NiFeOOH, it should be considered that the real active site may have bifunctional character, with separate roles for Ni and Fe. We will discuss these mechanisms in relation to a plausible mechanism for NiFeOOH further below. However, based on the reported data, we cannot provide definitive information on the active site, and therefore Figure 2 mainly serves an illustrative purpose.

Activation energy and pre-exponential factor over a larger potential range

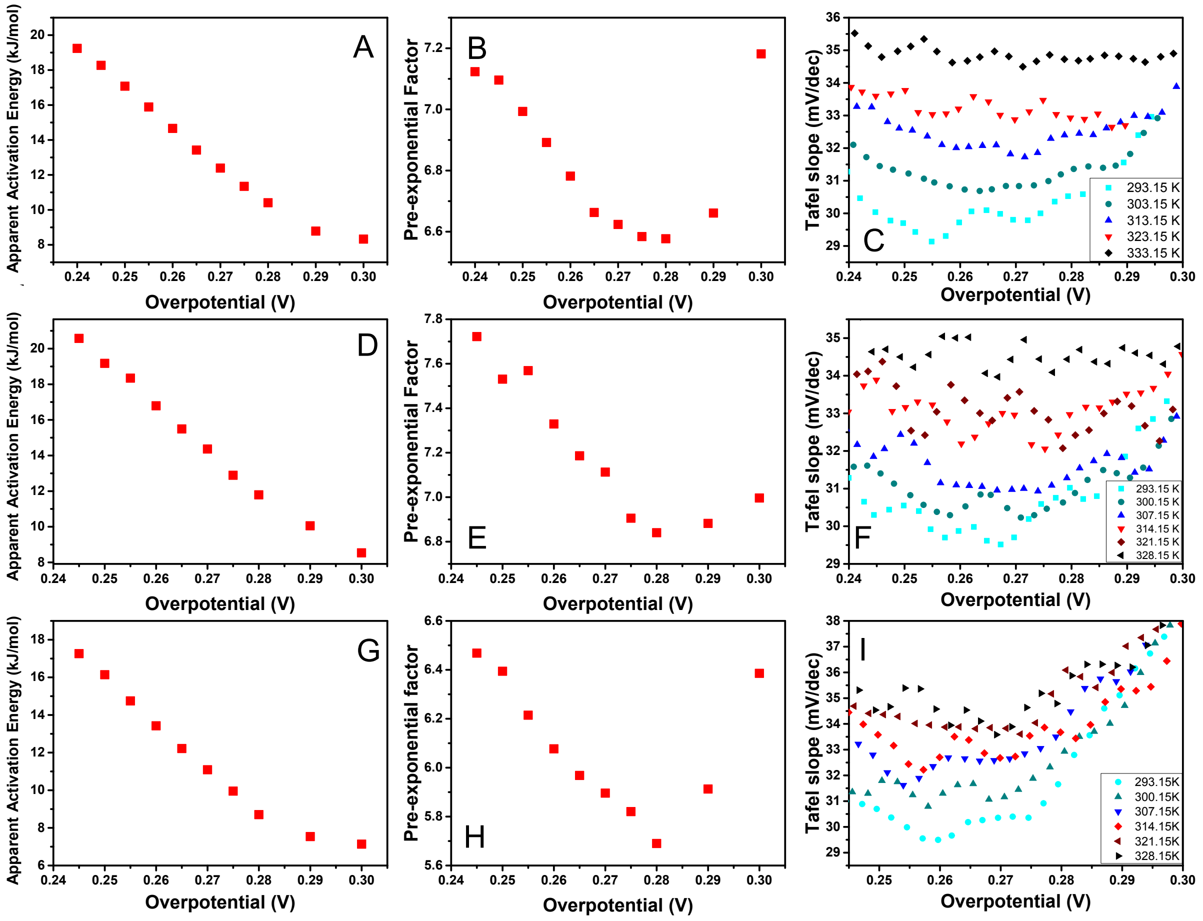

Figure S4. OER on NiFeOOH in 0.2 M KOH on a RDE setup at different temperatures ranging from 293.15 K to maximum 333.15 K, with the potential dependent activation energy (A, D, G), potential dependent pre-exponential factor (B, E, H) and temperature-dependent Tafel slope plots (C, F, I) of three different (but similar) measurements. The apparent activation energies and pre-exponential factors were determined from the plot of ln(i) at a given overpotential on the y-axis vs. 1/T on the x-axis, with the apparent activation energy being the slope multiplied by R and the pre-exponential factor the intercept with 1/T = 0, so extrapolated to “infinite temperature”.

Ni redox peaks

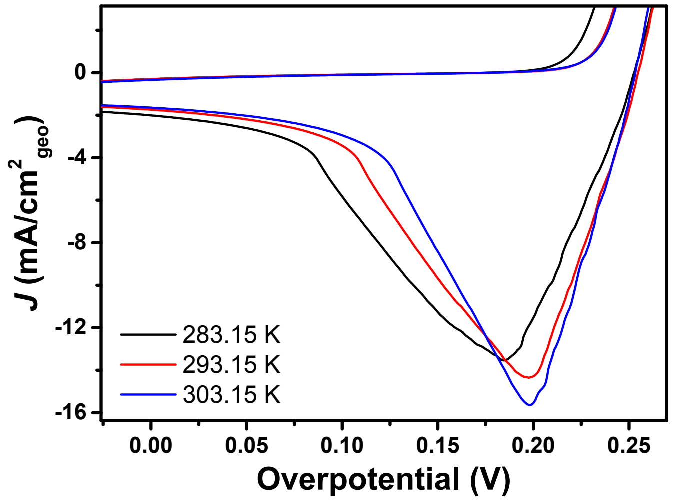

Figure S5. Ni reduction peak of NiFeOOH in 0.2 M KOH at 283.15, 293.15, 303.15 K vs. the overpotential at a scan rate of 50 mV/s. The reversibility of the Ni redox peak improves at higher temperatures, which is related to internal mass transport[17].

References

(1) E, P.; Kibsgaard, J.; Gallo, A.; Park, J.; Mitani, M.; Sokaras, D.; Kroll, T.; Sinclair, R.; Mogensen, M. B.; Jaramillo, T. F. Effects of Gold Substrates on the Intrinsic and Extrinsic Activity of High-Loading Nickel-Based Oxyhydroxide Oxygen Evolution Catalysts. *ACS Catal.* **2017**, *7* (8), 5399–5409. https://doi.org/10.1021/acscatal.7b01070.

(2) van der Heijden, O.; Eggebeen, J. J. J.; Trzesniowski, H.; Deka, N.; Golnak, R.; Xiao, J.; van Rijn, M.; Mom, R. V.; Koper, M. T. M. Li+ Cations Activate NiFeOOH for Oxygen Evolution in Sodium and Potassium Hydroxide. *Angew. Chemie Int. Ed.* **2024**, *63* (18), e202318692. https://doi.org/10.1002/anie.202318692.

(3) Mayrhofer, K. J. J.; Wiberg, G. K. H.; Arenz, M. Impact of Glass Corrosion on the Electrocatalysis on Pt Electrodes in Alkaline Electrolyte. *J. Electrochem. Soc.* **2008**, *155*, 1–5. https://doi.org/10.1149/1.2800752.

(4) Choudhury, D.; Das, R.; Maurya, R.; Kumawat, H.; Neergat, M. Kinetics of the Oxygen Evolution Reaction (OER) on Amorphous and Crystalline Iridium Oxide Surfaces in Acidic Medium. *Langmuir* **2023**, *39* (38), 13748–13757. https://doi.org/10.1021/acs.langmuir.3c02293.

(5) Trotochaud, L.; Young, S. L.; Ranney, J. K.; Boettcher, S. W. Nickel-Iron Oxyhydroxide Oxygen-Evolution Electrocatalysts: The Role of Intentional and Incidental Iron Incorporation. *J. Am. Chem. Soc.* **2014**, *136* (18), 6744–6753. https://doi.org/10.1021/ja502379c.

(6) van der Heijden, O.; Park, S.; Eggebeen, J. J. J.; Koper, M. T. M. Non-Kinetic Effects Convolute Activity and Tafel Analysis for the Alkaline Oxygen Evolution Reaction on NiFeOOH Electrocatalysts. *Angew. Chemie - Int. Ed.* **2023**, *62* (7), e202216477. https://doi.org/10.1002/anie.202216477.

(7) Kestin, J.; Sokolov, M.; Wakeham, W. A. Viscosity of Liquid Water in the Range -8 °C to 150 °C. *J. Phys. Chem. Ref. Data* **1978**, *7* (3), 941–948.

(8) Sonneveld, P. J.; Visscher, W.; Barendrecht, E. The Influence of Suspended Particles on the Mass Transfer at a Rotating Disc Electrode. Non-Conducting Particles. *J. Appl. Electrochem.* **1990**, *20* (4), 563–574. https://doi.org/10.1007/BF01008865.

(9) van der Heijden, O.; Park, S.; Vos, R. E.; Eggebeen, J. J. J.; Koper, M. T. M. Tafel Slope Plot as a Tool to Analyze Electrocatalytic Reactions. *ACS Energy Lett.* **2024**, *9*, 1871–1879. https://doi.org/10.1021/acsenergylett.4c00266.

(10) America, N. S. R. D. 69: N. C. W. U. S. S. of C. on behalf of the U. S. of. NIST Standard Reference Database 69: NIST Chemistry WebBook. **2023**.

(11) Bockris, J. O. M.; Otagawa, T. Mechanism of Oxygen Evolution on Perovskites. *J. Phys. Chem.* **1983**, *87* (15), 2960–2971. https://doi.org/10.1021/j100238a048.

(12) Wang, Z.; Goddard, W. A.; Xiao, H. Potential-Dependent Transition of Reaction Mechanisms for Oxygen Evolution on Layered Double Hydroxides. *Nat. Commun.* **2023**, *14* (1), 4228. https://doi.org/10.1038/s41467-023-40011-8.

(13) Marshall, A. T.; Vaisson-Béthune, L. Avoid the Quasi-Equilibrium Assumption When Evaluating the

Electrocatalytic Oxygen Evolution Reaction Mechanism by Tafel Slope Analysis. *Electrochem. commun.* **2015**, *61*, 23–26. https://doi.org/10.1016/j.elecom.2015.09.019.

(14) Mefford, J. T.; Zhao, Z.; Bajdich, M.; Chueh, W. C. Interpreting Tafel Behavior of Consecutive Electrochemical Reactions through Combined Thermodynamic and Steady State Microkinetic Approaches. *Energy Environ. Sci.* **2020**, *13* (2), 622–634. https://doi.org/10.1039/c9ee02697e.

(15) Baz, A.; Dix, S. T.; Holewinski, A.; Linic, S. Microkinetic Modeling in Electrocatalysis: Applications, Limitations, and Recommendations for Reliable Mechanistic Insights. *J. Catal.* **2021**, *404*, 864–872. https://doi.org/10.1016/j.jcat.2021.08.043.

(16) Govind Rajan, A.; Carter, E. A. Discovering Competing Electrocatalytic Mechanisms and Their Overpotentials: Automated Enumeration of Oxygen Evolution Pathways. *J. Phys. Chem. C* **2020**, *124* (45), 24883–24898. https://doi.org/10.1021/acs.jpcc.0c08120.

(17) Smith, R. D. L.; Sherbo, R. S.; Dettelbach, K. E.; Berlinguette, C. P. On How Experimental Conditions Affect the Electrochemical Response of Disordered Nickel Oxyhydroxide Films. *Chem. Mater.* **2016**, *28* (16), 5635–5642. https://doi.org/10.1021/acs.chemmater.6b01420.
